# Controversy in mechanotransduction – the role of endothelial cell–cell junctions in fluid shear stress sensing

**DOI:** 10.1242/jcs.262348

**Published:** 2024-09-09

**Authors:** Shaka X, Claire Aitken, Vedanta Mehta, Blanca Tardajos-Ayllon, Jovana Serbanovic-Canic, Jiayu Zhu, Bernadette Miao, Ellie Tzima, Paul Evans, Yun Fang, Martin A. Schwartz

**Affiliations:** ^1^ Yale Cardiovascular Research Center, Yale School of Medicine, New Haven, CT 06511, USA; ^2^ Cardiovascular Medicine, Radcliffe Department of Medicine, University of Oxford, Oxford OX3 7BN, UK; ^3^ Centre for Biochemical Pharmacology, William Harvey Research Institute, Barts and the London School of Medicine and Dentistry, Queen Mary University of London, London EC1M 6BQ, UK; ^4^ School of Medicine and Population Health, University of Sheffield, Sheffield S10 2RX, UK; ^5^ Department of Medicine, Biological Sciences Division, The University of Chicago, Chicago, IL 60637, USA; ^6^ Departments of Internal Medicine (Cardiovascular Medicine) and Cell Biology, Yale School of Medicine, and Biomedical Engineering, Yale University, New Haven, CT 06511, USA

**Keywords:** Endothelial, Cell junctions, Mechanosensation, Shear stress

## Abstract

Fluid shear stress (FSS) from blood flow, sensed by the vascular endothelial cells (ECs) that line all blood vessels, regulates vascular development during embryogenesis, controls adult vascular physiology and determines the location of atherosclerotic plaque formation. Although a number of papers have reported a crucial role for cell–cell adhesions or adhesion receptors in these processes, a recent publication has challenged this paradigm, presenting evidence that ECs can very rapidly align in fluid flow as single cells without cell–cell contacts. To address this controversy, four independent laboratories assessed EC alignment in fluid flow across a range of EC cell types. These studies demonstrate a strict requirement for cell–cell contact in shear stress sensing over timescales consistent with previous literature and inconsistent with the newly published data.

## Teaser

**Summary:** We demonstrate a strict requirement for cell–cell contact in shear stress sensing by endothelial cells over timescales consistent with previous literature and inconsistent with the newly published data.

## INTRODUCTION

Fluid shear stress (FSS) acting upon the vascular endothelium is required for crucial steps during vessel morphogenesis (Garcia-Cardena and Slegtenhorst, 2016; Roman and Pekkan, 2012) and for controlling blood flow and vessel remodeling in adult physiology (Baeyens et al., 2016; Lu and Kassab, 2011). Atherosclerotic plaques preferentially form at regions of lower and multidirectional FSS, termed disturbed FSS (Chatzizisis et al., 2007; Souilhol et al., 2020). One of the earliest observed response to physiological levels of FSS is the elongation and alignment of ECs in the direction of flow, which occurs over 6–24 h (Blackman et al., 2002; Dewey, 1984; Galbraith et al., 1998; Levesque and Nerem, 1985; van der Meer et al., 2010). By contrast, ECs fail to align in disturbed FSS, which has been identified as a causal factor in activation of the inflammatory pathways that drive atherogenesis (Wang et al., 2013).

Previous studies have found that endothelial cell (EC) alignment in flow required cell–cell contact and/or expression of cell–cell adhesion receptors, including PECAM-1, VE-cadherin, plexin D1 and latrophilin-2 (Conway et al., 2017; Masuda and Fujiwara, 1993; Mehta et al., 2020; Tzima et al., 2005; Tanaka et al., 2024). These receptors were also found to modulate flow-dependent vascular remodeling *in vivo* and development of atherosclerotic plaques (Chen and Tzima, 2009; Goel et al., 2008; Harry et al., 2008; Mehta et al., 2020; Stevens et al., 2008). Indeed, nanoparticles that target these molecules are under development for therapeutic applications (Khodabandehlou et al., 2017).

However, a recent high-profile publication cast some doubts upon these findings (Mylvaganam et al., 2022). This paper described a novel mechanism mediating the well-known elongation and alignment of vascular endothelial cells in the direction of fluid flow. Specifically, they reported that in an immortalized endothelial cell line, teloHAECs, alignment in the direction of fluid flow occurred over 30–60 min and occurred in sparse cultures where cells lacked cell–cell contacts. Given the importance of these issues to our understanding and treatment of vascular disease, we investigated the timing and cell contact dependence of flow-induced alignment in multiple EC types.

## RESULTS AND DISCUSSION

**Fig. 1.**
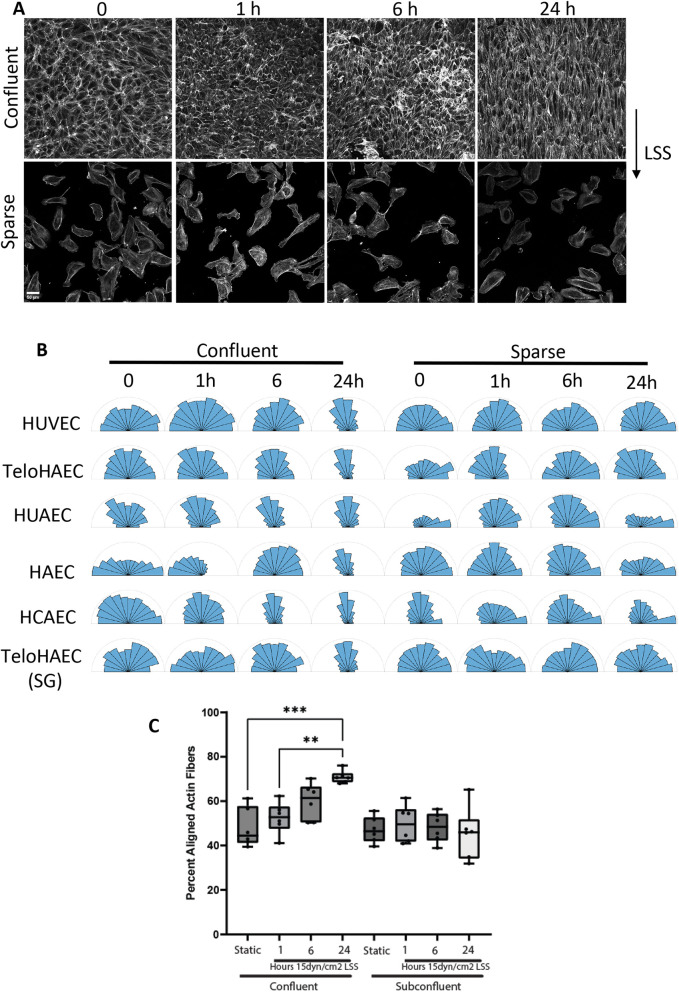
**Alignment of ECs in flow.** (A) HUVECs plated at confluent and sparse densities (100% and 10% confluence, respectively) were subject to laminar shear stress (LSS) at 15 dynes/cm^2^ for the indicated times, then cells were fixed, and F-actin was stained using Rhodamine–phalloidin. Scale bar: 50 μm. (B) Alignment in the direction of flow was quantified as described in the Materials and Methods. HAEC, human aortic ECs; HCAEC, human coronary artery ECs; TeloHAEC, (hTERT) immortalized HAECs (SG indicates the line obtained from the Grinstein lab); HUAEC, human umbilical artery ECs; HUVEC, human umbilical vein endothelial cells. *n*=3 independent experiments with >100 cells per condition for HUVECs and teloHAECs; *n*=1 with >50 cells per condition for HUAECs, HCAECs and teloHAEC from Sergio Grinstein (SG). (C) The percentage of aligned actin fibers was determined for each group, with 50% aligned being random. Each data point refers to an EC type from B. The box represents the 25–75th percentiles, and the median is indicated. The whiskers show the miniumum to maximum values. **P<0.005; ***P<0.005 [two-way ANOVA with Šídák's multiple comparisons test post test comparing conditions relative to static (without LSS)].

We set out to determine the time course and contact dependence for EC alignment in the direction of flow across a wide range of endothelial cell types. We examined human umbilical vein endothelial cells (HUVECs), human coronary artery endothelial cells (HCAECs), human aortic endothelial cells (HAECs) and the same teloHAEC line from ATCC used by Mylvaganam and co-workers (Mylvaganam et al., 2022). Cells were examined in confluent cultures and at low density using shear stress in the standard range of 12–15 dynes/cm^2^, similar to the 15 dynes/cm^2^ used in Mylvaganam et al. Confluent cells aligned over 6–24 h as expected, with no detectable alignment at 1 h. Single cells from the sparse cultures showed much less, if any, alignment over this period (Fig. 1). These results thus confirm the reported rate and cell–cell contact dependence of endothelial cell alignment across multiple endothelial cell types.

Dr Sergio Grinstein, the senior author in the Mylvaganam paper, kindly sent us the specific teloHAEC line used in his study and communicated details of the conditions used for the paper. However, we still see alignment over 12–24 h and no alignment in sparse cultures (Fig. 1B).

We conclude that all EC types tested align in flow over multiple hours and require cell–cell contacts. Thus, the results of Mylvaganam et al. cannot be replicated by experienced investigators in four independent laboratories. These new findings cement the current paradigm that junctional receptors play a central role in vascular responses to fluid flow, which clears the way for further progress in this area.

## MATERIALS AND METHODS

HUVECs were obtained from the Yale Vascular Biology and Therapeutics core facility. Each batch contains cells pooled from three deidentified donors. HUAECs, HAECs and HCAECs were obtained from promoc-cell and TeloHAECs from the ATCC. HUVECs, teloHAECs, HUAECs and teloHAECs (from Sergio Grinstein, Dept. of Biochemistry, Hospital for Sick Children, Toronto, Canada) were cultured in endothelial cell growth medium-2 with bullet kit (Lonza) and 5% FBS (Pinnacle Scientific, #PS-100) and were seeded on fibronectin-coated glass slides at 100% or 10% of confluent density (denoted confluent and sparse, respectively). Cells were subject to laminar shear stress at 15 dynes/cm^2^ using parallel plate flow chambers for the indicated and times. HAECs and HCAECs were cultured in endothelial cell growth medium MV2 plus supplements from Promocell (cat. no. C-22022; 0.05 ml/ml fetal calf serum, 5 ng/ml epidermal growth factor, 10 ng/ml basic fibroblast growth factor, 20 ng/ml insulin-like growth factor, 0.5 ng/ml vascular endothelial growth factor 165, 1 µg/ml ascorbic acid and 0.2 µg/ml hydrocortisone) plus 100 μg/ml streptomycin (Gibco), 100 U/ml penicillin (Gibco) and 2.5 μg/ml amphotericin B (Thermo Fisher Scientific). Cells were seeded on gelatin-coated 0.4 μm Ibidi^®^ slides at 10% or 100% confluency, and an Ibidi^®^ pump system was used for 12 dyn/cm^2^ shear stress for indicated times. All cells were cultured at 37°C and 95% humidity. For imaging, cells were fixed in 4% paraformaldehyde (ChemCruz, lot C0224), permeabilized (with 0.1% Triton X-100 in PBS), and stained for F-actin with Alexa Fluor 647-conjugated phalloidin (Thermo Fisher Scientific). Alignment in the direction of flow was quantified using a custom script which bins F-actin fibers by direction in 15° increments. This script is available at https://github.com/sudo-shaka/Actin-Alignment/.

HUVEC and teloHAEC rose plots combine counts across three independent replicates with >100 cells per condition. Other cell line data consists of one replicate with >50 cells per condition.

For each cell line and condition, the percentage of aligned actin fibers was calculated. Actin fibers were considered aligned if the orientation angle was <45° from the flow direction. Each point represents a cell line at that condition (Fig. 1C). Statistics were determined using two-way ANOVA with Šídák's multiple comparisons test post test comparing flow conditions and confluence, and is denoted **P<0.005; ***P<0.005.

## Supplementary Material



## References

[JCS262348C1] Baeyens, N., Bandyopadhyay, C., Coon, B. G., Yun, S. and Schwartz, M. A. (2016). Endothelial fluid shear stress sensing in vascular health and disease. *J. Clin. Invest.* 126, 821-828. 10.1172/JCI83083 26928035 PMC4767335

[JCS262348C2] Blackman, B. R., Garcia-Cardena, G. and Gimbrone, M. A., Jr (2002). A new in vitro model to evaluate differential responses of endothelial cells to simulated arterial shear stress waveforms. *J. Biomech. Eng.* 124, 397-407. 10.1115/1.1486468 12188206

[JCS262348C3] Chatzizisis, Y. S., Coskun, A. U., Jonas, M., Edelman, E. R., Feldman, C. L. and Stone, P. H. (2007). Role of endothelial shear stress in the natural history of coronary atherosclerosis and vascular remodeling: molecular, cellular, and vascular behavior. *J. Am. Coll. Cardiol.* 49, 2379-2393. 10.1016/j.jacc.2007.02.059 17599600

[JCS262348C4] Chen, Z. and Tzima, E. (2009). PECAM-1 is necessary for flow-induced vascular remodeling. *Arterioscler. Thromb. Vasc. Biol.* 29, 1067-1073. 10.1161/ATVBAHA.109.186692 19390054 PMC2723862

[JCS262348C5] Conway, D. E., Coon, B. G., Budatha, M., Arsenovic, P. T., Orsenigo, F., Wessel, F., Zhang, J., Zhuang, Z., Dejana, E., Vestweber, D. et al. (2017). VE-Cadherin phosphorylation regulates endothelial fluid shear stress responses through the polarity protein LGN. *Curr. Biol.* 27, 2219-2225.e2215. 10.1016/j.cub.2017.06.020 28712573 PMC5667920

[JCS262348C7] Dewey, C. F., Jr (1984). Effects of fluid flow on living vascular cells. *J. Biomech. Eng.* 106, 31-35. 10.1115/1.3138453 6539406

[JCS262348C8] Galbraith, C. G., Skalak, R. and Chien, S. (1998). Shear stress induces spatial reorganization of the endothelial cell cytoskeleton. *Cell Motil. Cytoskeleton* 40, 317-330. 10.1002/(SICI)1097-0169(1998)40:4<317::AID-CM1>3.0.CO;2-8 9712262

[JCS262348C9] Garcia-Cardena, G. and Slegtenhorst, B. R. (2016). Hemodynamic control of endothelial cell fates in development. *Annu. Rev. Cell Dev. Biol.* 32, 633-648. 10.1146/annurev-cellbio-100814-125610 27712101

[JCS262348C10] Goel, R., Schrank, B. R., Arora, S., Boylan, B., Fleming, B., Miura, H., Newman, P. J., Molthen, R. C. and Newman, D. K. (2008). Site-specific effects of PECAM-1 on atherosclerosis in LDL receptor-deficient mice. *Arterioscler. Thromb. Vasc. Biol.* 28, 1996-2002. 10.1161/ATVBAHA.108.172270 18669884 PMC3013511

[JCS262348C11] Harry, B. L., Sanders, J. M., Feaver, R. E., Lansey, M., Deem, T. L., Zarbock, A., Bruce, A. C., Pryor, A. W., Gelfand, B. D., Blackman, B. R. et al. (2008). Endothelial cell PECAM-1 promotes atherosclerotic lesions in areas of disturbed flow in ApoE-deficient mice. *Arterioscler. Thromb. Vasc. Biol.* 28, 2003-2008. 10.1161/ATVBAHA.108.164707 18688018 PMC2651147

[JCS262348C12] Khodabandehlou, K., Masehi-Lano, J. J., Poon, C., Wang, J. and Chung, E. J. (2017). Targeting cell adhesion molecules with nanoparticles using in vivo and flow-based in vitro models of atherosclerosis. *Exp. Biol. Med. (Maywood)* 242, 799-812. 10.1177/1535370217693116 28195515 PMC5407539

[JCS262348C14] Levesque, M. J. and Nerem, R. M. (1985). The elongation and orientation of cultured endothelial cells in response to shear stress. *J. Biomech. Eng.* 107, 341-347. 10.1115/1.3138567 4079361

[JCS262348C15] Lu, D. and Kassab, G. S. (2011). Role of shear stress and stretch in vascular mechanobiology. *J. R Soc. Interface* 8, 1379-1385. 10.1098/rsif.2011.0177 21733876 PMC3163429

[JCS262348C16] Masuda, M. and Fujiwara, K. (1993). Morphological responses of single endothelial cells exposed to physiological levels of fluid shear stress. *Front. Med. Biol. Eng.* 5, 79-87. 8241033

[JCS262348C17] Mehta, V., Pang, K. L., Rozbesky, D., Nather, K., Keen, A., Lachowski, D., Kong, Y., Karia, D., Ameismeier, M., Huang, J. et al. (2020). The guidance receptor plexin D1 is a mechanosensor in endothelial cells. *Nature* 578, 290-295. 10.1038/s41586-020-1979-4 32025034 PMC7025890

[JCS262348C18] Mylvaganam, S., Plumb, J., Yusuf, B., Li, R., Lu, C. Y., Robinson, L. A., Freeman, S. A. and Grinstein, S. (2022). The spectrin cytoskeleton integrates endothelial mechanoresponses. *Nat. Cell Biol.* 24, 1226-1238. 10.1038/s41556-022-00953-5 35817960

[JCS262348C19] Roman, B. L. and Pekkan, K. (2012). Mechanotransduction in embryonic vascular development. *Biomech. Model. Mechanobiol.* 11, 1149-1168. 10.1007/s10237-012-0412-9 22744845 PMC4502581

[JCS262348C20] Souilhol, C., Serbanovic-Canic, J., Fragiadaki, M., Chico, T. J., Ridger, V., Roddie, H. and Evans, P. C. (2020). Endothelial responses to shear stress in atherosclerosis: a novel role for developmental genes. *Nat. Rev. Cardiol.* 17, 52-63. 10.1038/s41569-019-0239-5 31366922

[JCS262348C21] Stevens, H. Y., Melchior, B., Bell, K. S., Yun, S., Yeh, J. C. and Frangos, J. A. (2008). PECAM-1 is a critical mediator of atherosclerosis. *Dis. Model. Mech.* 1, 175-181; discussion 179. 10.1242/dmm.000547 19048083 PMC2562188

[JCS262348C22] Tanaka, K., Chen, M., Prendergast, A., Zhuang, Z., Nasiri, A., Joshi, D., Hintzen, J., Chung, M., Kumar, A., Mani, A. et al. (2024). Latrophilin-2 mediates fluid shear stress mechanotransduction at endothelial junctions. *EMBO J.* 43, 3175-3191. 10.1038/s44318-024-00142-0 38886581 PMC11294477

[JCS262348C23] Tzima, E., Irani-Tehrani, M., Kiosses, W. B., Dejana, E., Schultz, D. A., Engelhardt, B., Cao, G., DeLisser, H. and Schwartz, M. A. (2005). A mechanosensory complex that mediates the endothelial cell response to fluid shear stress. *Nature* 437, 426-431. 10.1038/nature03952 16163360

[JCS262348C24] van der Meer, A. D., Poot, A. A., Feijen, J. and Vermes, I. (2010). Analyzing shear stress-induced alignment of actin filaments in endothelial cells with a microfluidic assay. *Biomicrofluidics* 4, 11103. 10.1063/1.3366720 20644662 PMC2905259

[JCS262348C25] Wang, C., Baker, B. M., Chen, C. S. and Schwartz, M. A. (2013). Endothelial cell sensing of flow direction. *Arterioscler. Thromb. Vasc. Biol.* 33, 2130-2136. 10.1161/ATVBAHA.113.301826 23814115 PMC3812824

