# Supplementary material for: Controversy in mechanotransduction – the role of endothelial cell–cell junctions in fluid shear stress sensing
Source: J Cell Sci. 2024 Sep 9;137(17):jcs262348. doi: 10.1242/jcs.262348 (PMC11423816; doi:10.1242/jcs.262348)
Supplement: Supplementary file 1 [file joces-137-262348_review_history.pdf]

Journal of Cell Science

Controversy in mechanotransduction: the role of endothelial cell-cell junctions in fluid shear stress sensing

Martin A. Schwartz, Shaka X, Claire Aitken, Vedanta Mehta, Blanca Tardajos Ayllon, Jovana Serbanovic-Canic, Jiayu Zhu, Bernadette Miao, Ellie Tzima, Paul Evans and Yun Fang
DOI: 10.1242/jcs.262348

Editor: Michael Way

Review timeline

Original submission:	12 June 2024
Editorial decision:	22 July 2024
First revision received:	7 August 2024
Accepted:	8 August 2024

Original submission

First decision letter

MS ID#: JOCES/2024/262348

MS TITLE: Controversy in mechanotransduction: the role of endothelial cell-cell junctions in fluid shear stress sensing

AUTHORS: Martin A. Schwartz, Shaka X, Claire Aitken, Vedanta Mehta, Blanca Tardajos Ayllon, Jovana Serbanovic-Canic, Jiayu Zhu, Bernadette Miao, Ellie Tzima, Paul Evans, and Yun Fang
ARTICLE TYPE: Short Report

I'm sorry this took so long but we have been waiting for the report from reviewer 2. The good news is we have now reached a decision on the above manuscript.

To see the reviewers' reports and a copy of this decision letter, please go to: https://submit-jcs.biologists.org and click on the 'Manuscripts with Decisions' queue in the Author Area. (Corresponding author only has access to reviews.)

As you will see, the reviewers gave favourable reports but raised some points requesting a few more details that will require amendments to your manuscript. I hope that you will be able to carry these out because I would like to be able to accept your paper.

Please ensure that you clearly highlight all changes made in the revised manuscript. Please avoid using 'Tracked changes' in Word files as these are lost in PDF conversion.

I should be grateful if you would also provide a point-by-point response detailing how you have dealt with the points raised by the reviewers in the 'Response to Reviewers' box. Please attend to all of the reviewers' comments. If you do not agree with any of their criticisms or suggestions please explain clearly why this is so.

Reviewer 1

Advance summary and potential significance to field

© 2024. Published by The Company of Biologists under the terms of the Creative Commons Attribution License (https://creativecommons.org/licenses/by/4.0/).

In this short but highly important study, Dr. Schwartz and three additional leaders in endothelial mechanotransduction address a major conceptual controversy in shear-induced cell alignment response. In a 2022 Nature Cell Biology paper, Dr. Grinstein reported that immortalized HAECs align with the direction of fluid shear stress and increase intracellular calcium and eNOS activation in a cell-cell contact-independent manner. They further elaborated on the cell-cell contract-independent mechanism involving spectrin, which orchestrates hyaluronic acid/glycocalyx with caveolae, piezo1, and eNOS responses.

Dr. Grinstein's finding that ECs can align with shear direction without requiring cell-cell contact was surprising and in conflict with prevailing prior reports by many groups, as the authors argue in this manuscript. The data carried out by 4 independent leading groups of endothelial mechanotransduction using 5 different EC types and the identical HTRT_HAEC used by Grinstein lab, as reported in this short manuscript, re-confirm that shear-induced alignment of ECs require cell-cell contact. The re-confirmation data directly and strongly challenges the claim by Dr. Grinstein's paper. While this reviewer agrees with the reconfirmation, it raises questions why and how?

As a side-note, it is my own lab's long-term finding that ECs (HAECs, HUVECs immortalized HAECs and immortalized MAECs) require at least 6hr to show any sign of cell alignment in (sub)confluent conditions. Dr. Grienstein's report that TERT-HAECs aligned with shear by 30 min in either confluent or sparse conditions has never been replicated in my lab.

Comments for the author

To help the readers, it would be helpful to provide some potential reasons for conflicting findings and details about experimental conditions between the groups including the different experimental conditions, shear device used, and shear duration.

Reviewer 2

Advance summary and potential significance to field

This letter organized by Schwartz and colleagues addresses the reproducibility of core observations in a 2022 publication (Mylvaganam et al) describing rapid endothelial cell alignment, and the alignment of single endothelial cells in response to applied fluid flow. This observation is fundamentally at conflict with robust prior research describing timescales of endothelial alignment under fluid shear stress, as well as the necessity of cell-cell contact and associated receptors in this process. Working across four labs and five endothelial cell lines, including the conditions and cells used in Mylvaganam et al., the authors do not reproduce the rapid alignment, and alignment of endothelial cells lacking cell-cell contact previously reported. The failure to reproduce these core observations is significant and altogether this letter is an important clarifying communication to the vascular biology community.

Comments for the author

I suggest the authors provide more detail in their methods rather than citing previous publication. In their reproduction of the previous paper's methods - “communicated details of the conditions used for the paper” – what were these conditions and were they different than those used for the other cell lines/assays?

- The cell line in question is referred to as TeloHAECs in the text, and hTert HAECs in the Figure and legend. I would choose one designation.
- Abstract fix: “While a number of papers that reported a…”
- Figure legend: HAAECs.
- Confluence is misspelled in the Figure.
- There is a “C” in the middle of the rose plots.

© 2024. Published by The Company of Biologists under the terms of the Creative Commons Attribution License (https://creativecommons.org/licenses/by/4.0/).

First revision

Author response to reviewers' comments

We thank the reviewers for the positive evaluation and helpful comments. We especially thank reviewer 1 for backing up our claims. ("Dr. Grinstein's report that TERT-HAECs aligned with shear by 30 min in either confluent or sparse conditions has never been replicated in my lab.")

Reviewer 1 Comments for the author
To help the readers, it would be helpful to provide some potential reasons for conflicting findings and details about experimental conditions between the groups, including the different experimental conditions, shear device used, and shear duration.

Good question. We went into this thinking we would find an explanation, most likely the Tert-HAEC cell line used in their studies. We also discussed their protocol with the PhD student who did the work (Mylvaganam). Their protocol was pretty much standard and none of the variables made any difference. On the other hand, both published data and the experiments done for this study using many different types of devices, different EC types and medium formulations have never reported alignment in 30-60 minutes or alignment of sparse cells. We simply do not have a clue and do not know what we could say that would be helpful.

Reviewer 2 Comments for the author
I suggest the authors provide more detail in their methods rather than citing previous publication. In their reproduction of the previous paper's methods - "communicated details of the conditions used for the paper" – what were these conditions and were they different than those used for the other cell lines/assays?
We have added more experimental detail to the Methods section.

- The cell line in question is referred to as TeloHAECs in the text, and hTert HAECs in the Figure and legend. I would choose one designation.
- Abstract fix: "While a number of papers that reported a…"
- Figure legend: HAAECs.
- Confluence is misspelled in the Figure.
- There is a "C" in the middle of the rose plots.

Thank you, we have corrected these errors.

Second decision letter

MS ID#: JOCES/2024/262348

MS TITLE: Controversy in mechanotransduction: the role of endothelial cell-cell junctions in fluid shear stress sensing

AUTHORS: Martin A. Schwartz, Shaka X, Claire Aitken, Vedanta Mehta, Blanca Tardajos Ayllon, Jovana Serbanovic-Canic, Jiayu Zhu, Bernadette Miao, Ellie Tzima, Paul Evans, and Yun Fang
ARTICLE TYPE: Short Report

I am happy to tell you that your manuscript has been accepted for publication in Journal of Cell Science, pending standard publication integrity checks.

© 2024. Published by The Company of Biologists under the terms of the Creative Commons Attribution License (https://creativecommons.org/licenses/by/4.0/).
